# Maintenance Therapy Containing Metformin and/or Zyflamend for Advanced Prostate Cancer: A Case Series

**DOI:** 10.1155/2015/471861

**Published:** 2015-03-15

**Authors:** Mehmet Asim Bilen, Sue-Hwa Lin, Dean G. Tang, Kinjal Parikh, Mong-Hong Lee, Sai-Ching J. Yeung, Shi-Ming Tu

**Affiliations:** ^1^Department of Genitourinary Medical Oncology, The University of Texas MD Anderson Cancer Center, Houston, TX 77030, USA; ^2^Department of Translational Molecular Pathology, The University of Texas MD Anderson Cancer Center, Houston, TX 77030, USA; ^3^Department of Molecular Carcinogenesis, The University of Texas MD Anderson Cancer Center, Houston, TX 77030, USA; ^4^Department of Molecular & Cellular Oncology, The University of Texas MD Anderson Cancer Center, Houston, TX 77030, USA; ^5^Department of Emergency Medicine, The University of Texas MD Anderson Cancer Center, Houston, TX 77030, USA

## Abstract

Metformin is derived from galegine, a natural ingredient, and recent studies have suggested that metformin could enhance the antitumor effects of hormone ablative therapy or chemotherapy and reduce prostate cancer-specific mortality. Zyflamend is a combination of herbal extracts that reduces inflammation and comprises turmeric, holy basil, green tea, oregano, ginger, rosemary, Chinese goldthread, hu zhang, barberry, and basil skullcap. We propose a maintenance regimen with metformin and/or Zyflamend that targets cancer stem cells and the tumor microenvironment to keep the cancer dormant and prevent it from activation from dormancy. Herein, we report the clinical course of four patients who experienced a clinical response after treatment with metformin and/or Zyflamend.

## 1. Introduction

The discovery over a half-century ago that prostate cancer depends on androgenic factors provided the foundation for an effective treatment for advanced prostate cancer [1]. Although hormone ablative therapy is effective, it is not a curative treatment. The unique relationship between prostate cancer and bone metastasis suggests that the missing link in a cure for prostate cancer may be in the bone and that maintenance therapy targeting the bone microenvironment could be a breakthrough for prostate cancer treatment.

Current treatment of bone metastasis in prostate cancer is inadequate. Hormone ablative therapy rarely produces complete remission in the osseous metastases of patients with advanced prostate cancer. Adding secondary treatments, such as an androgen-synthesis inhibitor (e.g., abiraterone), androgen-receptor antagonist (e.g., enzalutamide), chemotherapy (e.g., docetaxel, cabazitaxel), or cellular immunotherapy (e.g., sipuleucel-T) to hormone ablative therapy has improved the clinical outcome of patients with castration-resistant prostate cancer [2–6]. But the clinical benefit derived from these treatments is modest at best, with a median overall survival improvement of not more than 4 months.

Metformin is derived from galegine, a natural ingredient found in French lilac, Italian fitch, or Spanish sainfoin (*Galega officinalis*). Recent studies suggested that metformin could enhance the antitumor effects of hormone ablative therapy or chemotherapy and reduce prostate cancer-specific mortality [7–9]. Nondiabetic patients with prostate cancer who took metformin and adopted lifestyle changes had improved metabolic parameters [10]. Metformin is believed to act by selectively targeting prostate cancer stem cells, weakening transforming growth factor beta-induced epithelial-to-mesenchymal transition, and modulating an immune response and cancer-associated inflammation [11].

Zyflamend is a combination of herbal extracts comprising turmeric, holy basil, green tea, oregano, ginger, rosemary, Chinese goldthread, hu zhang, barberry, and basil skullcap. It reduces inflammation by inhibiting cyclooxygenase-2 and nuclear factor kappa B activity [12–14]. Zyflamend inhibited insulin-like growth factor-1-stimulated cell growth, insulin-like growth factor-1 receptor expression, and androgen-receptor expression and nuclear localization in a human castration-resistant prostate cancer cell line [15]. When given at human equivalent doses, it can potentiate the effects of androgen deprivation on tumor regression and growth inhibition of castration-sensitive and -resistant prostate cancers in vivo by reducing the expression of various biomarkers, including phosphorylated AKT and histone deacetylases [16].

Like other cancers, prostate cancer is a heterogeneous disease. However, unlike most other cancers, the bulk of differentiated prostate cancer is easily and well controlled with conventional treatments such as hormone ablative therapy. Using a maintenance regimen containing metformin and/or Zyflamend that targets putative prostate cancer stem cells and their obligate microenvironment may keep any minimal residual disease dormant and improve clinical outcome. Here, we report the clinical courses of four patients who experienced a clinical response to combination therapy with metformin and/or Zyflamend.

## 2. Case 1

**Figure 1:**
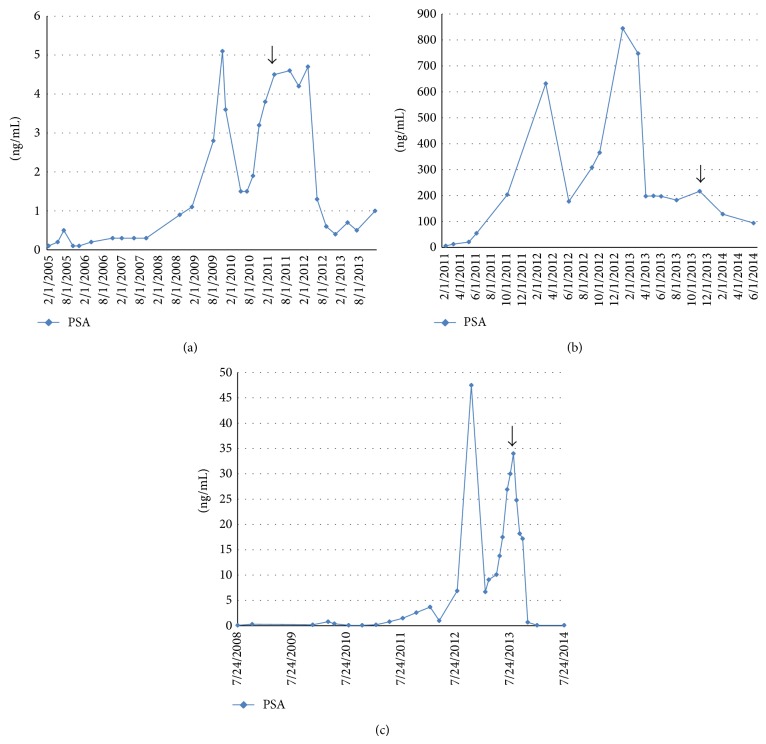
PSA plot for each patient and arrows indicate the initiation of combination therapy with metformin and/or Zyflamend (*x*-axis: time, *y*-axis: the value of PSA).

Case 1 was an 81-year-old African American man who was diagnosed with prostate cancer in 1974 and underwent radical prostatectomy. In 1987, he underwent salvage radiation therapy. Since 1994, he has received hormone ablative therapy, initially on an intermittent schedule. In September 2011, he received Zyflamend one tablet orally twice daily for his castration-resistant prostate cancer (arrow, Figure 1(a)). His prostate-specific antigen (PSA) level was increasing at an exponential rate to 4.5, and a bone scan was negative for metastasis. Zyflamend alleviated his arthritic pains, and his sense of well-being consequently improved. Surprisingly, his PSA level stabilized. More than a year later, his PSA decreased to 0.4 and remained stable for almost 2 years.

## 3. Case 2

Case 2 was a 60-year-old white man who was diagnosed with prostate cancer in January 2010. His PSA was 62, and his Gleason score was 9 (4 + 5). In March 2010, he underwent radical prostatectomy. His pathological disease stage was T3aN1. In July 2010, he started hormone ablative therapy. He had lymph node metastasis but no bone metastasis. In June 2011, he received sipuleucel-T. In January 2013, he received abiraterone. His PSA decreased from 844.2 to 182.5. In November 2013, his PSA increased to 216.6, and metformin 500 mg orally twice daily was added to the abiraterone. Surprisingly, his PSA decreased to 94.0 over the ensuing 6 months (arrow, Figure 1(b)).

## 4. Case 3

**Figure 2:**
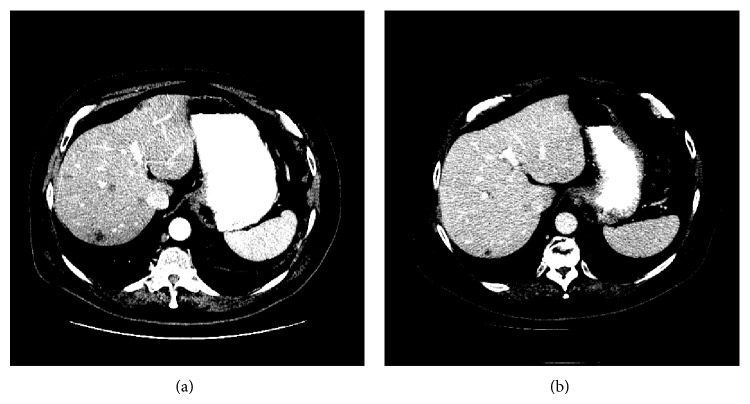
CT of the abdomen in case #3 revealed liver metastases (a) before the treatment and (b) after 17 months of treatment.

Case 3 was a 64-year-old white man who was diagnosed with prostate cancer in August 2007. His PSA was 93, his Gleason score was 9 (4 + 5), and he had pelvic lymphadenopathy at diagnosis. He received chemohormonal ablative therapy with weekly docetaxel at 25 mg/m^2^ followed by radical prostatectomy in September 2009. His pathological disease stage was T3aN0, and his surgical margins were negative. In November 2012, he developed recurrent lymphadenopathy and resumed hormone ablative therapy. In March 2013, he developed florid metastases in the liver, bone, lymph nodes, and bladder. Although his symptoms improved and his liver metastases and bladder lesions shrank, his PSA level continued to increase from 6.7 to 34.0 after 8 courses of salvage chemotherapy comprising cabazitaxel and carboplatin. In August 2013, metformin 500 mg orally twice daily was added to the chemotherapy regimen. Incredibly, his PSA decreased with the combined treatment (arrow, Figure 1(c)). CT findings of liver metastases and response to treatment are shown in Figure 2.

## 5. Case 4

Case 4 was a 76-year-old white man who was diagnosed with prostate cancer in 1994 and underwent radical prostatectomy. In 1995, he underwent salvage radiation therapy. In 1999, he received leuprolide and then bilateral orchiectomy. In October 2010, he developed recurrent disease in the prostatic bed, a biopsy of which showed adenocarcinoma with a Gleason score of 8 (4 + 4). From August 2011 until July 2013, he received chemotherapy comprising docetaxel and cabazitaxel and was then back to docetaxel again. Unfortunately, his PSA level continued to increase to 15.1. In August 2013, he discontinued chemotherapy and received a maintenance regimen comprising Zyflamend one tablet orally twice daily and metformin 500 mg orally twice daily. Incredibly, his PSA level decreased to 4.5 in November 2013 and was <0.1 in January 2014.

## 6. Discussion

Among the many important observations of cancer, dormancy is one of the most intriguing. Because dormancy is inherently multifactorial and integrated in nature (e.g., cell-cell interaction, microenvironment effect), it defies the traditional reductionist approach in our attempt to dissect it. How to harness cancer dormancy for therapeutic benefit is of interest. We proposed a maintenance regimen containing metformin and/or Zyflamend that targets cancer stem cells and the tumor microenvironment to keep the cancer dormant and prevent it from activating from dormancy.

It is well known that even the most curable cancers such as acute leukemia require some form of maintenance therapy to be cured [17]. Otherwise, for patients who had received no maintenance therapy after remission the median duration of remission was 4 months, and for all patients the disease relapsed. Unfortunately, attaining a complete remission in solid tumors is a rare feat. To the best of our knowledge, establishing the clinical value of maintenance therapy in solid tumors has never been accomplished.

According to the stem cell theory of cancer, treatments that target cancer stem cells have a special place in the maintenance therapy of cancer [18, 19]. When it is not feasible to eradicate cancer stem cells, it may still be feasible to control them with maintenance therapy. Ideally, this task is best accomplished in the setting of minimal residual disease, when we have the best chance to keep the tumor dormant or indolent by manipulating the tumor microenvironment, if not the tumor itself. In this regard, maintenance therapy for prostate cancer is particularly well suited because of the efficacy of conventional treatments, such as hormone ablative therapy.

Warburg first reported altered metabolism and increased aerobic glycolysis that is maintained in conditions of high oxygen tension leading to enhanced lactate production in cancer [20]. It turns out that cancer cells preferentially utilize glycolysis despite fully functional mitochondria [21]. Metformin's multiple mechanisms of action on various molecular, cellular, and microenvironment targets suggest that it may provide clinical benefits by promoting cancer dormancy and synergizing with the antitumor effects of conventional treatments [22–29].

In many respects, the observation if not the idea of dormancy contradicts the genetic theory of cancer. After all, genetic mutations are less likely to occur and unlikely to be selected in the absence of any cellular growth, division, or activity [30]. Because dormancy is an intrinsic property of stem cells and certain somatic cells, it may not be necessary to invoke any specialized genes or specific mutations (i.e., “reinventing the wheel”) for those cells to be induced into or released from dormancy.

Currently, clinical trials are not designed to assess the therapeutic effects and potential benefits of agents that target cancer stem cells or the tumor microenvironment; furthermore, treatments that target cancer stem cells or the tumor microenvironment may provide difficult-to-measure delayed effects and therapeutic benefits. Consequently, promising treatments could be prematurely abandoned unless and until we develop therapeutic strategies to account for the presence of cancer stem cells and the tumor microenvironment as well as response criteria to monitor the effects of therapy on them.

The suggestion that metformin and/or Zyflamend exert antitumor effects beyond androgen pathways is illuminating and refreshing. Of note, all four patients who responded to the treatment had castration-resistant prostate cancer. By targeting prostate cancer stem cells and the tumor microenvironment, metformin and/or Zyflamend could provide an alternative if not novel means of treating prostate cancer in general and castration-resistant prostate cancer in particular.

Importantly, any maintenance treatment, if proven to be efficacious, will need to be taken by patients for a prolonged period of time. It is imperative that we discover and demonstrate that such treatments are safe and affordable. Natural products like metformin and/or Zyflamend that provide multipronged mechanisms of action seem well matched against the multifaceted nature of cancer. However, more basic and clinical research needs to be performed before patients become empowered by an effective, safe, and affordable maintenance regimen for the treatment of prostate cancer.

## 7. Conclusion

Here, we report the clinical course of 4 patients who experienced a clinical response to combination therapy with metformin and/or Zyflamend. By targeting prostate cancer stem cells and the tumor microenvironment, metformin and/or Zyflamend could provide an alternative if not novel means of treating prostate cancer and enhance the therapeutic benefit of conventional treatments.
